# Genome-wide QTL mapping of nine body composition and bone mineral density traits in pigs

**DOI:** 10.1186/s12711-014-0068-2

**Published:** 2014-10-28

**Authors:** Sophie Rothammer, Prisca V Kremer, Maren Bernau, Ignacio Fernandez-Figares, Jennifer Pfister-Schär, Ivica Medugorac, Armin M Scholz

**Affiliations:** Chair of Animal Genetics and Husbandry, Ludwig-Maximilians-University Munich, Veterinärstrasse 13, 80539 Munich, Germany; Department Agrikultur, University of Applied Sciences Weihenstephan-Triesdorf, Weidenbach, Germany; Livestock Center of the Faculty of Veterinary Medicine, Ludwig-Maximilians-University Munich, Oberschleissheim, Germany; Spanish National Research Council (CSIC), Granada, Spain

## Abstract

**Background:**

Since the pig is one of the most important livestock animals worldwide, mapping loci that are associated with economically important traits and/or traits that influence animal welfare is extremely relevant for efficient future pig breeding. Therefore, the purpose of this study was a genome-wide mapping of quantitative trait loci (QTL) associated with nine body composition and bone mineral traits: absolute (Fat, Lean) and percentage (FatPC, LeanPC) fat and lean mass, live weight (Weight), soft tissue X-ray attenuation coefficient (R), absolute (BMC) and percentage (BMCPC) bone mineral content and bone mineral density (BMD).

**Methods:**

Data on the nine traits investigated were obtained by Dual-energy X-ray absorptiometry for 551 pigs that were between 160 and 200 days old. In addition, all pigs were genotyped using Illumina’s PorcineSNP60 Genotyping BeadChip. Based on these data, a genome-wide combined linkage and linkage disequilibrium analysis was conducted. Thus, we used 44 611 sliding windows that each consisted of 20 adjacent single nucleotide polymorphisms (SNPs). For the middle of each sliding window a variance component analysis was carried out using ASReml. The underlying mixed linear model included random QTL and polygenic effects, with fixed effects of sex, housing, season and age.

**Results:**

Using a Bonferroni-corrected genome-wide significance threshold of *P* < 0.001, significant peaks were identified for all traits except BMCPC. Overall, we identified 72 QTL on 16 chromosomes, of which 24 were significantly associated with one trait only and the remaining with more than one trait. For example, a QTL on chromosome 2 included the highest peak across the genome for four traits (Fat, FatPC, LeanPC and R). The nearby gene, *ZNF608*, is known to be associated with body mass index in humans and involved in starvation in Drosophila, which makes it an extremely good candidate gene for this QTL.

**Conclusions:**

Our QTL mapping approach identified 72 QTL, some of which confirmed results of previous studies in pigs. However, we also detected significant associations that have not been published before and were able to identify a number of new and promising candidate genes, such as *ZNF608*.

**Electronic supplementary material:**

The online version of this article (doi:10.1186/s12711-014-0068-2) contains supplementary material, which is available to authorized users.

## Background

Domestication of the pig took place more than 9000 years ago. Since then, natural and artificial selection have led to the formation of many different breeds and strains. Currently, the pig is one of the most important livestock animals with more than 250 breeds worldwide. While most breeds (e.g. Large Black, Schwäbisch-Hällisch and Cerdo Iberico) serve mainly for commercial niche (high price) markets, for large-scale commercial pork production, only a few breeds (e.g. Large White, Landrace, Pietrain, Duroc and Hampshire) are used [1,2]. These major breeds are of enormous economic interest and thus, it is not surprising that much effort has been put into Quantitative Trait Locus (QTL) mapping in pigs during the last two decades (reviewed by [1,3]). Today, there are almost 10 000 QTL listed in the Pig QTL database (pigQTLdb, [4]; SS_10.2 downloaded on 04.02.2014). Among other reasons, insufficient resolution due to marker availability has limited traditional QTL analyses (many QTL cover more than 20 cM), which has delayed identification of causal genes or variants [5]. However, recently, the availability of cost-effective genome-wide SNP genotyping has made it possible to map QTL more accurately and this has increased opportunities to identify candidate genes and/or to implement genomic selection. During the last years, the first genome-wide association studies using Illumina’s Porcine SNP60 Genotyping Beadchip were published (e.g. [6-10]). On the one hand, pig mapping data are important for long-term efficient pig breeding that integrates animal welfare. On the other hand, the pig is an extremely relevant animal model since it shares many genetic and physiological similarities with humans [11]. Thus, candidate regions/genes that are mapped for distinct traits in the pig can also be important for similar traits in humans and vice versa. In particular, loci associated with fatness and fat composition in pig can be relevant for the study of human obesity and obesity-associated diseases that are some of the most severe health issues [6,8,12,13].

In this study, we carried out a genome-wide (44 809 SNPs) combined linkage and linkage-disequilibrium analysis (LDLA) in a dataset of 551 pigs that were a mixture of the following breeds in various proportions: Large Black, Pietrain, Duroc, Schwäbisch-Hällisch, Cerdo Iberico, European Wild boar, and Hampshire. The whole body traits that were analyzed included four traits for body composition (Fat g, FatPC %, Lean g, LeanPC %), three traits for bone mineral content and density (BMC g, BMCPC %, BMD g/cm^2^), the soft tissue X-ray attenuation coefficient (R) and live weight (Weight g). Except for Weight, all traits were obtained by Dual-energy X-ray absorptiometry (DXA), as described in Scholz and Förster [14] and in Kremer et al. [15]. Although candidate gene analyses for leg weakness and bone mineral traits have been performed at local levels (e.g. [16-18]), to our knowledge, this is the first genome-wide QTL mapping analysis for whole body bone mineral traits in pigs. Bone mineral traits are of special interest in pig production since they have been shown to be associated with osteochondrosis [17] and bone fracture risk [16]. Thus, these traits are part of the multifactorial leg weakness syndrome which is a serious animal welfare issue and moreover causes considerable economic losses [18-20]. Significant results (genome-wide *P*-value < 0.001) of the genome-wide QTL mapping of these nine traits will be important for pig breeding. In addition, we identified QTL for Fat and FatPC that might represent regions of special interest for future analysis of obesity-related traits in humans.

## Methods

### Ethical statement

The trials reported in this work comply with the ethical guidelines of the Ludwig-Maximilians-University (LMU) of Munich and were conducted with the approval of the appropriate ethics committee of the District Government of Upper Bavaria, Germany (No. 55.2-1-54-2532.2-60-07 and No. 55.2-1-54-2531.2-22-08). Moreover, blood sampling was conducted only by certified veterinarians who follow the German Animal Welfare Act to avoid any unnecessary pain, suffering and damage to the animals.

### Animal samples

For this study, 554 blood samples of individuals between 160 and 200 days old were collected at the Livestock Center of the Faculty of Veterinary Medicine (Oberschleissheim, Germany). The animals sampled represented six different breeds (Duroc, Cerdo Iberico, Large Black, Hampshire, Pietrain, Schwäbisch-Hällisch) and included both purebred and crossbred individuals. In addition to these six breeds, crossbred animals included varying proportions (0 to 25%) of European Wild boar.

For all individuals, weight was measured with a mechanic livestock scale for weights between 1 and 250 kg and eight body composition traits were measured by dual energy X-ray absorptiometry (DXA) using a pencil beam scanner “GE Lunar DPX-IQ” with the whole body mode “adult normal” (Lunar software version 4.7e, GE Healthcare, Pittsburgh, PA, USA), as described in [14,15]. Values were predicted for bone mineral content in g (BMC) and percentage (BMCPC), bone mineral density in g/cm^2^ (BMD), fat tissue mass in g (Fat) and percentage (FatPC), lean tissue mass in g (Lean) and percentage (LeanPC), and the X-ray attenuation coefficient (R). Prior to DXA-scanning, animals were fasted for 16 hours. They were sedated by intramuscular injection of azaperone (Stresnil®: 1.2 mg/kg body weight) followed by ketamine (Ursotamin®: 40 mg/kg body weight) and then, an intravenous catheter was placed in an ear vein to continue ketamine administration if necessary.

### Genotyping data and quality control

DNA extraction from blood samples was performed using the QIAamp DNA Blood Mini Kit from Qiagen. DNA of all sampled pigs was genotyped for 62 163 single nucleotide polymorphisms (SNPs) using version 1 of the PorcineSNP60 Genotyping BeadChip (Illumina Inc., San Diego, USA). The physical positions of all SNPs were downloaded according to the reference assembly SGSC Sscrofa10.2/susScr3 [21]. Markers that met one of the following criteria were excluded from further analysis: (i) successful genotyping results in less than 95% of the animals, (ii) frequent paternity conflicts in animals with known paternity, (iii) unknown or non-unique position according to the reference assembly susScr3, (iv) markers with a heterozygosity level less than 0.05 and (v) markers on porcine chromosomes X and Y since we concentrated on autosomes only. After this filtering process, the marker dataset comprised 44 809 markers.

From the initial animal set, three individuals were excluded from further analysis because of a genome-wide genotyping call rate below 95% resulting in a final animal set of 551 animals.

### Reconstruction of haplotypes

For haplotype reconstruction and imputation of missing genotypes, the Hidden Markov Model implemented in BEAGLE 3.0.4 was used [22]. Since additional animals and pedigree information improve the accuracy of haplotype reconstruction, we added another 49 animals that were otherwise not relevant for this analysis, and assigned all animals to three different groups: (i) 169 parent-offspring trios, (ii) 251 parent-offspring pairs and (iii) 151 unrelated individuals without any genotyped parent or offspring. It should be noted that some animals can be offspring in a pair and parent in a trio. Thus, the sum of all individuals in pairs, trios and the unrelated group exceeded the actual number of animals in the dataset.

### Unified additive relationships and principal component analysis

To correct for population stratification and family relationships within the mixed linear model used for QTL mapping (see below), we estimated the unified additive relationships (UAR) between all animals [23]. Subsequently, all principal components of the UAR matrix were estimated using R [24]. The number of principal components to be integrated in the linear model was determined using the R package paran [25], which is an implementation of the empirical method of Horn’s parallel analysis. Based on the so-defined 18 principal components, we were able to efficiently reduce the initial matrix dimensions from 551×551 to 18×551, without significant loss of information since these 18 principal components explained more than 90% of the genetic variance.

### Locus IBD and diplotype relationship matrix

Besides genome-wide relationships, local haplotype relationships were also included in the mixed linear model for QTL mapping. Genome-wide relationships were accounted for by using the 18 most significant principal components of the UAR matrix, while for local haplotype relationships we used sliding windows of 20 consecutive SNPs along the genome (larger windows of e.g. 40 SNPs also performed well and showed very similar results to the 20-SNP windows; data not shown). At each window-midpoint (i.e. between markers 10 and 11), the locus identity by descent (LocIBD) was estimated based on the method described in Meuwissen and Goddard [26]. The resulting haplotype-based IBD matrices were then converted into diplotype relationship matrices (**D**_**RM**_) using the procedure described for additive genetic relationship matrices at a QTL (**G**_**RM**_ matrix) in Lee and Van der Werf [27].

### Genome-wide QTL mapping

QTL mapping was carried out by a procedure that is equivalent to the combined linkage/linkage disequilibrium mapping method reported in Meuwissen et al. [28]. While linkage information was accounted for during reconstruction of haplotypes based on available relationships, linkage disequilibrium is considered in the estimation of LocIBD. Finally, a variance component analysis in the middle of each of the 20-SNP sliding windows was performed in ASReml [29]. The mixed linear model included random QTL effects based on **D**_**RM**_, as well as the fixed effects of sex, housing, season, and the covariates of age at sampling and the 18 principal components to account for polygenic effects. Effects of additional corrections, such as pedigree-based breed compositions, were also tested, but since they were not significant, they were omitted from the final mixed linear model. The resulting model was:

$$ \mathbf{y}=\mathbf{X}\boldsymbol{\upbeta } +\mathbf{Z}\mathbf{q}+\mathbf{e}, $$

where **y** is a vector of phenotypes of the investigated trait, **β** a vector of fixed effects (including overall mean *μ*, fixed effects, age at sampling and the 18 principal components), **q** a vector of random additive genetic effects due to QTL with **q** ~ N(0,**D**_**RMp**_${\sigma}_q^2$), where **D**_**RMp**_ is the diplotype relationship matrix at position *p* of the putative QTL and **e** a vector of random residual effects with **e** ~ N(0,**I**${\sigma}_e^2$), where **I** is an identity matrix.

The random effects **q** and **e** were assumed to be uncorrelated and normally distributed and their variances (${\sigma}_q^2$, ${\sigma}_e^2$) were simultaneously estimated using ASReml [29].

Using the logarithm of the likelihood estimated by ASReml for the model with (logL_P_) and without QTL effects (logL_0_; corresponding to the null hypothesis), we calculated the likelihood ratio test statistic (LRT = −2(logL_0_-logL_P_)), which is known to be *Χ*^2^-distributed with one degree of freedom [30]. In order to keep the false discovery rate low, we chose a conservative significance threshold of LRT = 31.275, which corresponds to a *P*-value less than 2.24*10^−8^ before correction for multiple-testing and a Bonferroni-corrected genome-wide *P*-value less than 0.001 based on 44 611 tested windows (0.001/44 611 = 2.24*10^−8^). For each peak that exceeded this LRT threshold, we determined the confidence interval (CI) of the position of the QTL using the 2-LOD (log of odds; 1 LOD = 4.605 LRT) drop-off criterion [5,31]. Overlapping CI of neighboring peaks for the same trait were combined into a single CI. In such cases, only the peak with the highest LRT was reported for the combined CI. Overlapping CI for different traits were assigned to a common QTL. For all genes within a QTL, a literature study was conducted to identify the QTL’s candidate gene(s). As candidates we considered genes that fulfilled one of the following criteria: (i) genes within QTL for the same or related traits identified in previous studies in pig, (ii) known to be associated with related traits in other mammals or species and (iii) involved in pathways related to the investigated trait.

## Results

### Genome-wide QTL mapping

**Figure 1:**
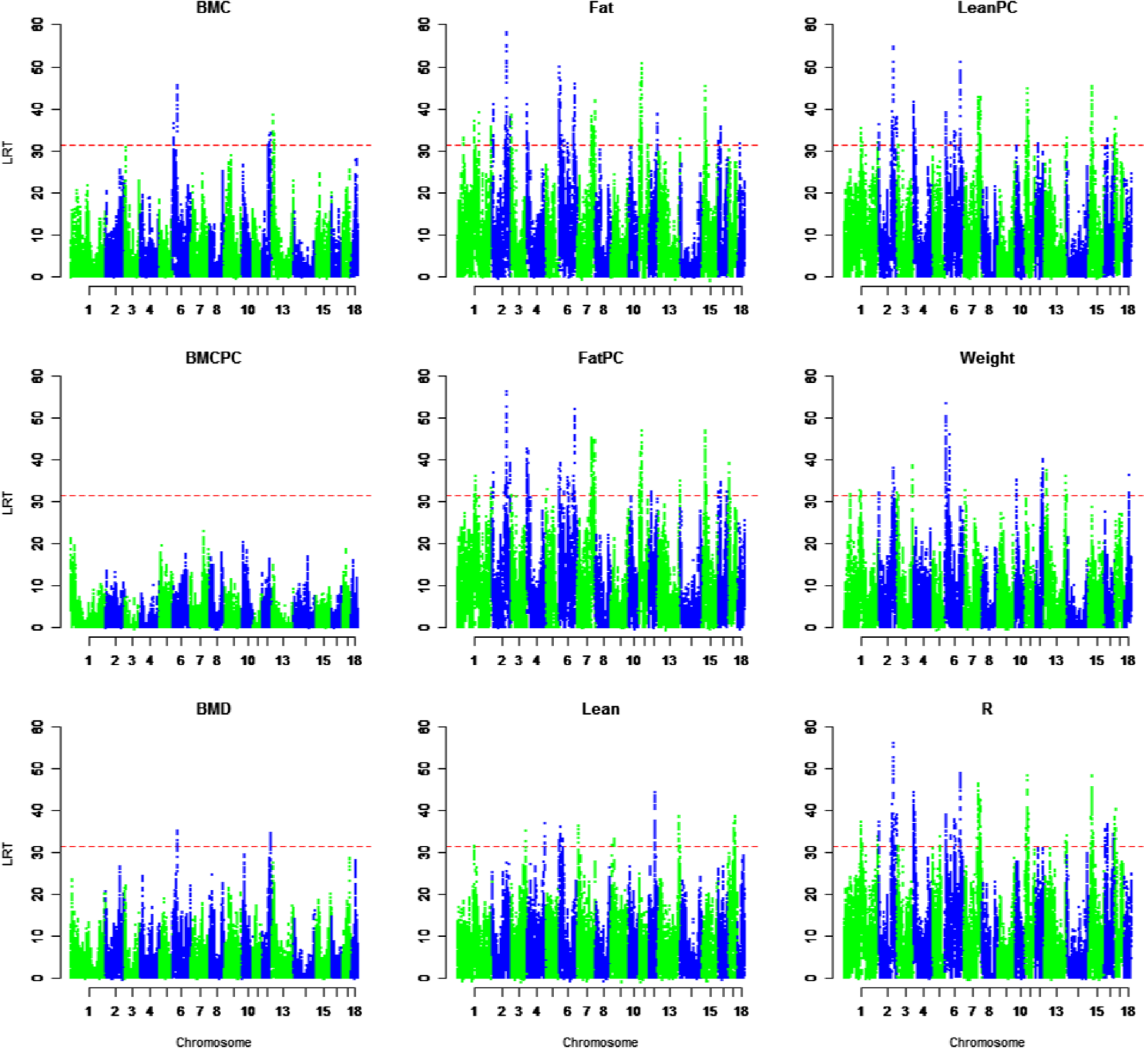
**Manhattan plot of QTL-mapping results.** LRT-values (y-axes) are shown for all nine traits and autosomes (x-axes); a red dashed line marks the genome-wide significance threshold of LRT = 31.275 (*P* < 0.001).

As shown in Figure 1, significant associations of distinct chromosomal regions with phenotype were found for each of the nine traits investigated, except for BMCPC. However, the number of identified QTL varied considerably from 2 for BMD to 41 for FatPC. Chromosomes 8 and 14 showed no significant associations while the remaining chromosomes had up to 31 (SSC6) significant associations. Table 1 shows a summary of the significant associations and their confidence intervals (CI) identified per trait and chromosome. A total of 195 associations were identified for all traits, which represented 72 QTL. The CI of only 24 of the 195 associations did not overlap with the CI for another trait (See Additional file 1).

**Table 1:** **Number of significant associations by trait and chromosome**

**Chr**	**BMC**	**BMD**	**Fat**	**FatPC**	**Lean**	**LeanPC**	**Weight**	**R**	**Sum**
1	-	-	5	5	1	3	2	5	21
2	-	-	6	6	-	5	3	6	26
3	-	-	1	1	2	-	4	1	9
4	-	-	3	3	1	3	-	3	13
5	-	-	-	1	-	-	-	1	2
6	3	1	5	5	3	5	4	5	31
7	-	-	5	6	1	4	1	6	23
8	-	-	-	-	-	-	-	-	0
9	-	-	-	-	2	-	-	-	2
10	-	-	-	-	-	-	1	-	1
11	-	-	5	4	-	3	-	3	15
12	3	1	1	1	1	1	2	-	10
13	2	-	1	1	1	1	2	1	9
14	-	-	-	-	-	-	-	-	0
15	-	-	3	3	-	4	-	3	13
16	-	-	2	3	-	1	-	3	9
17	-	-	-	2	3	2	-	2	9
18	-	-	1	-	-	-	1	-	2
Total	8	2	38	41	15	32	20	39	195

Based on overlaps of CI between traits, two QTL stood out, i.e. one QTL on chromosome 6 between 7 006 410 and 14 176 920 bp and one QTL on chromosome 2 between 129 544 104 and 135 064 176 bp. Within the region on chromosome 6, the CI of seven traits (BMC, Fat, FatPC, Lean, LeanPC, R, Weight) overlapped and Weight had its highest LRT-value across the genome (53.279) in this region at position 8 723 052. The region on chromosome 2 included five significant traits (Fat, FatPC, LeanPC, R, Weight). Each of these traits, except Weight, had its highest LRT-values across the genome (54.78-58.1) in this region and at exactly the same position (133 237 232 bp).

Peak-positions and CI of the highest genome-wide association for each trait are in Table 2. While BMC and BMD shared the highest peak at the same position (37 403 520 bp) in another region of chromosome 6, the greatest LRT for Lean (44.279) was on chromosome 12 (42 949 544 bp). Table 2 also lists candidate genes for each QTL. These candidates and additional interesting candidate genes will be discussed in the next section.

**Table 2:** **Highest peak identified across the genome for each trait**

**Trait**	**Chr**	**Peak**	**LRT**	**CI Start**	**CI End**	**Mb**	**Candidate genes**
Fat	2	133 237 232	58.1	129 544 104	135 064 176	5.52	ZNF608
FatPC	2	133 237 232	56.16	129 544 104	135 064 176	5.52	ZNF608
LeanPC	2	133 237 232	54.78	129 544 104	135 064 176	5.52	ZNF608
R	2	133 237 232	55.82	129 544 104	135 064 176	5.52	ZNF608
Weight	6	8 723 052	53.279	7 334 450	9 257 070	1.92	(WWOX)
BMC	6	37 403 520	45.56	36 937 640	37 714 128	0.78	- (TSHZ3)
BMD	6	37 403 520	35.14	36 937 640	37 714 128	0.78	- (TSHZ3)
Lean	12	42 949 544	44.279	41 217 576	45 554 716	4.34	chemokines, NF1

Genes in parenthesis lie outside of the respective confidence interval (CI).

## Discussion

It has been shown that a meta-analysis that combines data from multiple populations improves both power and resolution of QTL mapping (reviewed by [3]). This raises the obvious question of whether combining multiple populations in a single-QTL mapping study will also provide more significant results. As for F2 populations in line-cross models [3], animal datasets that contain diverse populations and crosses should allow the mapping of QTL that are fixed in a population. In this paper, we show that diverse animal datasets such as the one used in this study can confirm many previously published QTL and, moreover, identify new unknown QTL.

Overall, we found that most of the identified QTL (48 out of 72) affected more than one trait, which is not surprising since the traits studied are not completely independent. While relationships of Fat or Lean with the percentage traits FatPC and LeanPC, as well as with Weight are obvious, there is also a linear relationship between FatPC and ratio of the mass attenuation coefficients of soft tissue (R value = X-ray attenuation coefficient) [32-34]. Moreover, it has been extensively shown in humans that obesity correlates with bone metabolism (reviewed by [35,36]). We will come back to these relations when discussing possible candidate genes (full names of all gene symbols mentioned hereafter are in Additional file 2).

It is worth mentioning that some of the mapped QTL lie in the vicinity of an even stronger QTL for the same trait(s). In such cases, further investigations, such as multiple-QTL analyses, are needed to clarify if these are separate QTL or rather represent carryover effects of nearby large QTL [37,38]. Nevertheless, for several of these “possible carryover QTL”, there is some indication that they are separate QTL since promising candidate genes could be defined within the CI of some QTL. For example on chromosome 7, there are seven non-overlapping CI for Fat and/or FatPC (referred to as CI1 to CI7 in the following) within a segment of 31 Mb (between 99 352 936 and 130 971 280 bp). Although, some of these could be caused by carryover effects, we were able to identify candidate genes within each CI, some of which were reported in previous QTL studies in pigs or were associated with functions related to body composition in pigs and other mammals: (i) *SIPA1L1* and *DPF3* are good candidates for CI1 (between 99 352 936 and 102 221 456 bp) since the chromosomal segment containing these genes was reported to be associated with 10^th^ rib backfat in pig [39]; (ii) for CI2 (between 104 522 416 and 105 871 296 bp), *TGFB3* was identified as a candidate gene since it may be involved in adipogenesis [40]; (iii) *DIO2* is a candidate gene for CI3 (between 106 921 984 and 109 413 032 bp) because *Dio2* knock-out mice were shown to be prone to obesity [41]; (iv) *GALC* was defined as a candidate gene for CI4 (between 110 964 904 and 116 355 960 bp) because it is part of a fine-mapped QTL (~3 Mb) for intramuscular fat content [42]; (v) for CI5 (between 117 489 560 and 120 450 760 bp), we identified *TTC7B* as a promising candidate gene because it was reported to be a candidate gene for obesity in mice [43]; (vi) for CI6 (between 121 429 704 and 124 338 264), the genes *PRIMA1, FAM181A, ASB2, OTUB2, DDX24* and *ISG12(A)* overlapped with a chromosomal region that was reported to be associated with 10^th^ rib backfat by Fan et al. [39]; and (vii) for CI7 (between 125 575 968 and 130 971 280 bp), we identified *DIO3* as a promising candidate gene since it was found to be associated with a number of fat deposition and carcass traits in pig [44]. However, without further analysis it is not possible to discriminate carryover effects from individual nearby QTL.

### Candidate genes for the highest peaks across the genome

All traits except BMCPC reached a genome-wide significance of *P* < 0.001. The eight highest peaks across the genome (one for each significant trait) belonged to four QTL on three chromosomes (Table 2). The highest peaks of Fat (LRT = 58.1), FatPC (56.16), LeanPC (54.78) and R (55.82) were assigned to one of these QTL. Moreover, an additional significant peak for Weight (LRT = 37.899) fell within this confidence interval (Chr2:129 544 104–135 064 176). Since the peak positions for Weight, at 133 150 512 bp, and for the other traits, at 133 237 232 bp, were only separated by two markers, it can be assumed that all significant associations identified in this region share a common mechanism. The gene that is closest to both peaks according to the Ensembl genome browser [45] is *ZNF608,* located between 133 256 788 and 133 396 887 bp. *ZNF608* has been reported to be associated with body mass index (BMI) in humans [46,47]. Moreover, scribbler (sbb), which is the *ZNF608* gene homologue in Drosophila [48], is suggested to be involved in larval food search behavior under starvation conditions [49]. Although the molecular mechanisms that underlie the function of *ZNF608* are not clear [48], it seems to play a fundamental role in related traits across species. Therefore, *ZNF608* can be regarded as a candidate gene associated with body composition in pigs. Since the five traits that have significant associations in this region are not independent and Fat has the highest LRT while the non-significant trait Lean had the lowest LRT among all traits for this region except BMCPC, it can be hypothesized that *ZNF608* influences directly absolute fat mass and thereby indirectly the remaining traits (FatPC, LeanPC, R and Weight).

The highest peak for Weight (LRT = 53.279) was observed at the beginning of chromosome 6 at 8 723 052 bp. The CI for this association (between 7 334 450 and 9 257 070 bp) included significant peaks for six other traits: Lean (LRT = 36.0), BMC (36.6), R (38.8), LeanPC (38.92), FatPC (39.1) and Fat (49.84). The genes closest to the peak based on NCBI Map Viewer are *CDYL2* between 7 681 404 and 7 695 761 bp at the proximal end and, in addition to a *5S ribosomal RNA* gene (between 9 222 444 and 9 222 549 bp), two genes near the distal end: *CLEC3A* between 9 640 139 and 9 647 945 bp and *WWOX* between 9 684 708 and 9 744 573 bp. Differential expression between human osteoarthritic and normal cartilage has been reported for *CLEC3A* [50] and *WWOX* [51], which indicates an association with bone formation. Thus, *CLEC3A* and *WWOX* are promising candidate genes for BMC. Moreover, *WWOX*-deficient (−/−) mice die three weeks post-partum and display multiple postnatal defects, such as growth retardation and abnormalities in bone metabolism [52]. Therefore, it can be hypothesized that *WWOX* variants could also affect growth in pigs and, thus, traits such as Weight, Lean, LeanPC, FatPC, Fat and the fat-related R.

Another QTL on chromosome 6 had its highest peak across the genome at 37 403 520 bp for BMC (45.56) and BMD (35.14). At exactly the same position, Weight had a significant peak with an even higher LRT-value (45.76) than BMC and BMD. The CI for this QTL, from 36 937 640 to 37 714 128 bp, has no annotated gene, neither in Ensembl nor in the NCBI Map Viewer [53]. However, according to Ensembl, the two nearest genes are less than 150 and 30 kb apart from the CI position, i.e. *TSHZ3* between 36 787 929 and 36 791 170 bp and *ZNF507* between 37 742 417 and 37 767 697 bp. No known information about *ZNF507* function suggests that it could be a candidate gene for BMC or BMD, but *TSHZ3* has been shown to be associated with both muscle differentiation [54] and lipid traits [55]. Thus, one hypothesis for this QTL could be that, assuming that both Fat and Lean are affected only moderately by a *TSHZ3* variant in the same direction, there is no significant effect on either trait but the accumulated effects are significant for Weight. Since weight is known to influence bone mineral traits [34], a significant change in Weight might also have significant effects on BMD and BMC. For example, Scholz et al. [34] found a positive correlation of 0.85 between soft tissue mass and BMD (*in vivo*: adjusted R^2^ = 0.72).

The highest peak for Lean (44.279) was identified on chromosome 12 at 42 949 544 bp. Within the CI for this association (between 41 217 576 and 45 554 716 bp), we also detected significant peaks for BMC (31.28 at 42 637 020 bp) and Weight (35.439 at 42 365 148 bp). The three peaks are spread over a region that contains 33 markers and includes the genes *CCL1* and *CCL11*, *CCL2* and *CCL8*, the novel protein coding gene *ENSSSCG00000030066* and one pseudogene (*ENSSSCG00000017724*). For a number of chemokines, among which are *CCL2*, *CCL8* and *CCL11*, increased expression has been demonstrated in adipose tissue of obese human patients. Thus, it was hypothesized that chemokines might be involved in promoting adipose tissue inflammation in obesity [56]. Moreover, a relationship between higher serum levels of the inflammatory chemokines *CCL11* and *CCL2* with lower lean body mass has been shown in geriatric patients [57]. *NF1* is another interesting candidate gene within the CI of the peak for Lean. This gene is involved in neurofibromatosis type 1 in humans. Among other complications, affected individuals are likely to have low BMD, which is probably caused by *NF1* haploinsufficiency [58,59]. Moreover, *NF1* seems to be essential for muscle development and metabolism, because *NF1* muscle-specific knock-out mice weigh significantly less than control mice [58]. Although *NF1* lies within the CI of a peak for Lean only, it is a remarkable candidate gene that could be directly involved in not only Lean but also Weight and BMC.

### Additional candidate genes

Besides the candidate genes mentioned in the previous section, we were able to define at least one candidate gene for most (49 out of 72) of the QTL. For reasons of clarity, we will concentrate only on some of these candidate genes in the following, but in Additional files 3, 4, 5, 6, 7, 8, 9 and 10, all candidate genes are marked in bold letters.

#### QTL associated with Fat, FatPC, LeanPC and R traits

The QTL region on chromosome 6 for Fat, FatPC, LeanPC and R spans more than 5 Mb (between 133 183 304 and 138 202 064 bp). To date, this region contains more than 40 annotated genes based on Ensembl and NCBI Map Viewer. Among these, eight genes were considered as candidate genes for the associated traits: *DIRAS3, PDE4B, LEPROT,* (*LEPR*)*, DNAJC6, AK3L1, JAK1* and *PGM1*. For *DIRAS3,* a correlation of the expression level with intramuscular fat has been confirmed in cattle [60]. The sub-region that contains *PDE4B*, *LEPROT*, *DNAJC6*, *AK3L1* and *JAK1* has been associated with backfat thickness in the Duroc pig breed [61] and a segment that includes *LEPROT*, *DNAJC6,* and *AK3L1* was declared to be associated with intramuscular fat content in pigs [62]. *PDE4B* has been associated with backfat thickness in pigs and with obesity in humans [7,62,63]. In addition, Lee et al. [64] detected significant associations with backfat thickness for both *PDE4B* and *LEPROT*. Although to date *LEPR* has not been positioned on the Sscrofa 10.2 assembly, it is assumed to be located next to *LEPROT* based on the human genome map and thus within the QTL region. For *LEPR*, significant associations with average daily weight gain and backfat thickness in Duroc pigs have been demonstrated [65]. Further evidence for a relation between *LEPR* and fat-related traits is available from the literature, i.e. a highly significant differential expression of *LEPR* in muscle tissue of pig breeds that display divergent obesity traits was reported by [66] and an association between a non-coding variation in *LEPR* and higher BMI was demonstrated in Native Americans [67]. Although the localization of *LEPR* within our QTL region needs to be confirmed, it represents a promising candidate gene, especially for fat-related traits. Another candidate gene in this QTL region, *PGM1*, has been shown to be significantly associated with BMI in humans [68]. Moreover, higher expression of *PGM1* was detected in fast-growing compared to slow-growing chickens [69]. Overall, this QTL spans a chromosomal region that has been shown in many studies to be involved with, in particular, fat-related traits in pigs, cattle, humans, and mice. However, without further investigations we cannot narrow down the number of candidate genes or assess a possible interaction between several of these candidate genes. Thus, deciphering the cause of a QTL might be much easier for QTL that contain only few candidate genes. Some examples of such QTL will be discussed in the following.

#### QTL associated with Weight

Within a 2 Mb QTL for Weight on chromosome 1, between 107 871 792 and 109 893 944 bp, which contains 20 genes, we identified two possible candidate genes: *LIPG* and *ACAA2*. In humans, LIPG expression was shown to be increased in obese individuals compared to normal weight individuals [70]. Therefore, we hypothesize that *LIPG* may also be involved in lipid metabolism pathways in the pig and affect Weight. *ACAA2*, the second candidate gene, encodes an enzyme that catalyzes the last step of the mitochondrial fatty acid beta-oxidation spiral [71]. Thus, since *ACAA2* is also involved in the pathway of fat metabolism, it may affect Weight.

#### QTL associated with FAT

Another 1.5 Mb QTL that spans almost 20 annotated genes was detected for Fat on chromosome 1 (between 38 634 124 and 40 136 908 bp). A possible candidate gene for this QTL is *RSPO3* since loci near this gene were reported to be associated with waist-hip ratio in humans with European [72] or African ancestry [73].

#### QTL associated with FatPC and R

We mapped a 1.5 Mb QTL for FatPC and R (between 104 522 416 and 105 871 296 bp) on chromosome 7. *TGFB3* is the most promising candidate among the 13 genes in this chromosomal region based on results of Li et al. [74] who demonstrated an association between a polymorphism in the *TGFB3* gene and growth and body composition traits in chickens, including weight of breast muscle and abdominal fat pad. Thus, it can be hypothesized that *TGFB3* variants influence both fat and lean mass, which may cause a significant change in FatPC and consequently in R.

#### QTL associated with FatPC and LeanPC

A QTL for FatPC and LeanPC that extended over less than 500 kb was detected on chromosome 12 (between 14 714 488 and 15 183 214 bp). We identified *GH1* as an obvious candidate gene for this QTL since Fontanesi et al. [75] reported a significant association of a marker within the 3’-UTR region of *GH1* with backfat thickness in the Italian Large White pig breed. In addition, a premature stop codon in *GH1* causes an increased adipose tissue accumulation in zebrafish among other abnormalities (such as decreased somatic growth), which supports the importance of *GH1* for fat-related traits, even across species [76].

#### Comparison of our results with those of Fan et al.

Finally, we develop some reflections on our results in the light of the findings of Fan et al. [39] since we detected numerous chromosomal regions that they had identified in their GWA study on body composition in pigs. Five of the chromosomal regions that Fan et al. [39] found to be associated with last rib backfat or 10^th^ rib backfat match QTL that we mapped for Fat or FatPC. Two other regions reported by Fan et al. [39] overlap with QTL for Lean and another one with a QTL for Weight. It should also be noted that three candidate chromosomal regions detected for body length, hip structure, and weak top line overlap with QTL for Weight in our study. All genes that overlap with regions reported by Fan et al. [39] are marked by asterisks in Additional files 3, 4, 5, 6, 7, 8, 9 and 10. A region that was associated with last rib backfat on chromosome 2 in Fan et al. [39] overlapped with one of our QTL for Fat, FatPC, LeanPC, Weight and, R (between 7 012 572 and 9 118 948 bp). For this region, Fan et al. [39] did not suggest a candidate gene. Interestingly, annotation of our QTL with the currently known genes revealed two genes, *RCOR2* (between 7 350 357 and 7 352 786 bp) and *MARK2* (between 7 355 161 and 7 364 707 bp), within this region that were not reported by Fan et al. [39], which suggests that these genes were annotated in the pig genome only recently. Hurov et al. [77] showed that a loss of the MARK2 polarity kinase leads to decreased adiposity. No association with appropriate traits has been established for *RCOR2*. Thus, *MARK2* is a candidate gene for both the QTL detected in our study and the region associated with last rib backfat thickness in Fan et al. [39].

## Conclusions

Our LDLA-based QTL mapping approach revealed significant peaks for all traits except for BMCPC. Most of the 72 identified QTL were associated with more than one trait and some of these associations confirmed previously reported results in pigs. However, we also detected significant associations in regions that have not been published before. Based on the literature, we identified candidate genes for most of the detected QTL and suggest a number of new and promising candidate genes, such as *ZNF608* or *WWOX*.

## Additional files

Additional file 1:
**195 confidence intervals for 72 QTL.** Description: For each of the 72 mapped QTL (consecutively numbered) the respective significant phenotype (PT), its peak position (Peak) in bp (chromosome: Chr) and the maximal LRT-value (LRT) are given. In addition, start (CI start in bp) and end (CI end in bp) of each confidence interval and its size in Mb are given. QTL that are framed in red are those for which more than one PT was significant. Additional file 2:
**Gene names.** Description: This table provides the full names of all gene symbols mentioned within the main text. Additional file 3:
**Genome-wide confidence intervals for BMC.** Description: The consecutive QTL number based on Additional file 1 is given for each confidence interval (CI) as well as chromosome, peak-position with respective LRT-value, CI position in bp and distance in Mb. Genes listed in the last column are all known, characterized genes according to Ensembl and NCBI genome browsers. Genes in bold characters are candidate genes that are based on a literature search for related traits and pathways in the pig and other species. Genes that were shown to be associated with related traits in Fan et al. [39] are marked with asterisks. Additional file 4:
**Genome-wide confidence intervals for BMD.** Description: The consecutive QTL number based on Additional file 1 is given for each confidence interval (CI) as well as chromosome, peak-position with respective LRT-value, CI position in bp and distance in Mb. Genes listed in the last column are all known, characterized genes according to Ensembl and NCBI genome browsers. Genes in bold characters are candidate genes that are based on a literature search for related traits and pathways in the pig and other species. Genes that were shown to be associated with related traits in Fan et al. [39] are marked with asterisks. Additional file 5:
**Genome-wide confidence intervals for Fat.** Description: The consecutive QTL number based on Additional file 1 is given for each confidence interval (CI) as well as chromosome, peak-position with respective LRT-value, CI position in bp and distance in Mb. Genes listed in the last column are all known, characterized genes according to Ensembl and NCBI genome browsers. Genes in bold characters are candidate genes that are based on a literature search for related traits and pathways in the pig and other species. Genes that were shown to be associated with related traits in Fan et al. [39] are marked with asterisks. Additional file 6:
**Genome-wide confidence intervals for FatPC.** Description: The consecutive QTL number based on Additional file 1 is given for each confidence interval (CI) as well as chromosome, peak-position with respective LRT-value, CI position in bp and distance in Mb. Genes listed in the last column are all known, characterized genes according to Ensembl and NCBI genome browsers. Genes in bold characters are candidate genes that are based on a literature search for related traits and pathways in the pig and other species. Genes that were shown to be associated with related traits in Fan et al. [39] are marked with asterisks. Additional file 7:
**Genome-wide confidence intervals for Lean.** Description: The consecutive QTL number based on Additional file 1 is given for each confidence interval (CI) as well as chromosome, peak-position with respective LRT-value, CI position in bp and distance in Mb. Genes listed in the last column are all known, characterized genes according to Ensembl and NCBI genome browsers. Genes in bold characters are candidate genes that are based on a literature search for related traits and pathways in the pig and other species. Genes that were shown to be associated with related traits in Fan et al. [39] are marked with asterisks. Additional file 8:
**Genome-wide confidence intervals for LeanPC.** Description: The consecutive QTL number based on Additional file 1 is given for each confidence interval (CI) as well as chromosome, peak-position with respective LRT-value, CI position in bp and distance in Mb. Genes listed in the last column are all known, characterized genes according to Ensembl and NCBI genome browsers. Genes in bold characters are candidate genes that are based on a literature search for related traits and pathways in the pig and other species. Genes that were shown to be associated with related traits in Fan et al. [39] are marked with asterisks. Additional file 9:
**Genome-wide confidence intervals for Weight.** Description: The consecutive QTL number based on Additional file 1 is given for each confidence interval (CI) as well as chromosome, peak-position with respective LRT-value, CI position in bp and distance in Mb. Genes listed in the last column are all known, characterized genes according to Ensembl and NCBI genome browsers. Genes in bold characters are candidate genes that are based on a literature search for related traits and pathways in the pig and other species. Genes that were shown to be associated with related traits in Fan et al. [39] are marked with asterisks. Additional file 10:
**Genome-wide confidence intervals for R.** Description: The consecutive QTL number based on Additional file 1 is given for each confidence interval (CI) as well as chromosome, peak-position with respective LRT-value, CI position in bp and distance in Mb. Genes listed in the last column are all known, characterized genes according to Ensembl and NCBI genome browsers. Genes in bold characters are candidate genes that are based on a literature search for related traits and pathways in the pig and other species. Genes that were shown to be associated with related traits in Fan et al. [39] are marked with asterisks.
